# Hemoglobin A1c in early pregnancy to identify preexisting diabetes mellitus and women at risk of hyperglycemic pregnancy complications

**DOI:** 10.1016/j.xagr.2024.100315

**Published:** 2024-01-19

**Authors:** Ka Wang Cheung, Tiffany Sin-Tung Au, Chi-Ho Lee, Vivian Wai Yan Ng, Felix Chi-Kin Wong, Wing-Sun Chow, Pui Wah Hui, Mimi Tin Yan Seto

**Affiliations:** 1 Department of Obstetrics and Gynaecology, Queen Mary Hospital, The University of Hong Kong, Hong Kong, China (Dr Cheung, Ms Au, Dr Ng, Dr Hui, and Dr Seto); 2 Department of Medicine, Queen Mary Hospital, School of Clinical Medicine, The University of Hong Kong, Hong Kong, China (Dr Lee and Dr Chow); 3 Department of Pathology, Queen Mary Hospital, Hong Kong, China (Dr Wong)

**Keywords:** complication, gestational diabetes, hyperglycemia, iron deficiency, prediabetes, thalassemia

## Abstract

**BACKGROUND:**

Unrecognized diabetes mellitus during pregnancy could pose serious maternal and neonatal complications. A hemoglobin A1c level of ≥6.5% was used to diagnose both diabetes mellitus in nonpregnant individuals and diabetes in pregnancy. As the hemoglobin A1c level could be influenced by maternal physiological changes, the optimal cutoff in early pregnancy to detect women with diabetes in pregnancy and associated complications remains unclear.

**OBJECTIVE:**

This study aimed to evaluate the diagnostic performance of various hemoglobin A1c levels and the optimal hemoglobin A1c cutoff to identify mothers with diabetes in pregnancy diagnosed by the gold standard 75 g oral glucose tolerance test before 24 weeks of gestation. In addition, the pregnancy and neonatal outcomes were compared using the optimal hemoglobin A1c cutoff.

**STUDY DESIGN:**

A retrospective cohort study was conducted between 2004 and 2019. Women with at least 1 risk factor of gestational diabetes mellitus received an oral glucose tolerance test before 24 weeks of gestation. Terminology of hyperglycemia first detected during pregnancy by oral glucose tolerance test was classified as either diabetes in pregnancy or gestational diabetes mellitus following the World Health Organization's recommendation. Women who met the diagnostic criteria of diabetes in pregnancy and early-onset gestational diabetes mellitus (ie, before 24 weeks of gestation) and had a paired hemoglobin A1c measurement within 4 weeks of their early oral glucose tolerance test were studied. Sensitivity, specificity, and positive and negative predictive values at various hemoglobin A1c cutoffs were calculated for the detection of diabetes in pregnancy. The optimal hemoglobin A1c level was identified from the constructed receiver operating characteristic curves. Multivariate binary logistic regression analyses were performed to calculate the unadjusted and adjusted odds ratios for pregnancy complications.

**RESULTS:**

There were 63,111 deliveries, and 22,949 women underwent an oral glucose tolerance test before 24 weeks of gestation. A total of 157 and 3210 women met the diagnostic criteria of diabetes in pregnancy and early-onset gestational diabetes mellitus using an oral glucose tolerance test, respectively. Only 346 participants had a paired hemoglobin A1c and oral glucose tolerance test measurement (82 cases with diabetes in pregnancy and 264 cases with early-onset gestational diabetes mellitus). The receiver operating characteristic curve identified an optimal hemoglobin A1c cutoff of 5.7% to diagnose diabetes in pregnancy, with a sensitivity of 64.6%, specificity of 81.1%, positive predictive value of 51.5%, and negative predictive value of 88.1%. A hemoglobin A1c cutoff of either 5.9% or 6.5% could miss 47.6% or 73.2% of women with diabetes in pregnancy. In multivariate logistic regression analysis, a hemoglobin A1c level of ≥5.7% increased the risk of maternal insulin use (adjusted odds ratio, 6.69; 95% confidence interval, 3.44–12.99), macrosomia (adjusted odds ratio, 7.43; 95% confidence interval, 1.90–29.00), and shoulder dystocia (adjusted odds ratio, 6.56; 95% confidence interval, 1.161–37.03).

**CONCLUSION:**

The optimal hemoglobin A1c cutoff to detect diabetes in pregnancy diagnosed using an oral glucose tolerance test before 24 weeks of gestation was 5.7%, but this cutoff could not reliably identify diabetes in pregnancy owing to the low sensitivity. However, an early hemoglobin A1c level of ≥5.7% indicated increased risks of pregnancy and neonatal complications.

**AJOG Global Reports at a Glance**

**Why was this study conducted?**

Hemoglobin A1c (HbA1c) in early pregnancy may identify women at risk of diabetes in pregnancy (DIP) and associated complications, but the appropriate cutoff remains unclear.

**Key findings**

The optimal cutoff of HbA1c to diagnose DIP in early pregnancy was 5.7%, but this had a low sensitivity of 64.6%. Women with an HbA1c level of ≥5.7% in early pregnancy were at risk of maternal insulin use, macrosomia, and shoulder dystocia.

**What does this add to what is known?**

HbA1c may be incorporated as part of the routine antenatal screening, and an HbA1c level of 5.7% could identify women at risk of hyperglycemic pregnancy complications.

## Introduction

The prevalence of type 2 diabetes mellitus (T2D) increased rapidly from 108 million to 422 million between 1980 and 2014.^1^ Unrecognized preexisting diabetes mellitus during pregnancy could pose serious maternal and neonatal complications, for example, congenital defects, preterm birth, stillbirth, large for gestational age, shoulder dystocia, preeclampsia, birth trauma, and cesarean delivery.^2^ Diabetes in pregnancy (DIP) (or overt diabetes mellitus) is defined as hyperglycemia first detected during pregnancy, which meets the diagnostic criteria of T2D.^3^^,^^4^ As most women may not have proper screening before pregnancy, DIP is a commonly used terminology to represent pregnant women with possible preexisting diabetes mellitus. Delaying detection until routine screening at 24 to 28 weeks of gestation for gestational diabetes mellitus (GDM) could miss the opportunities for earlier identification and intervention. The American Diabetes Association suggested using either random glucose, oral glucose tolerance test (OGTT), or hemoglobin A1c (HbA1c) before 15 weeks of gestation to diagnose DIP if women were not screened before pregnancy.^5^ However, the ideal method to reveal unrecognized preexisting T2D in early pregnancy remained unknown.^4^

In nonpregnant individuals, HbA1c is commonly used to diagnose T2D and monitor the level of glycemic control. In pregnancy, HbA1c could estimate the risk of congenital fetal anomalies in women with preexisting T2D.^6^ Compared with OGTT, which is the conventional test recommended to screen for and diagnose GDM,^3^ HbA1c is more convenient and acceptable because it does not require overnight fasting and only 1 venipuncture is needed; hence, it could be readily incorporated into the routine antenatal screening program during early pregnancy. An HbA1c level of ≥6.5% was recommended to detect DIP during pregnancy, and the same cutoff was used to diagnose T2D in nonpregnant individuals.^7^ However, the value of HbA1c could be influenced by hemoglobinopathy, the degree of erythropoiesis, erythrocyte destruction, and glycation.^8^ Moreover, several maternal physiological changes, such as increased red blood cell turnover, could lead to lower HbA1c levels, whereas iron deficiency, which is common during pregnancy, could elevate HbA1c levels.^9^ Therefore, using the same HbA1c cutoff value aiming at identifying women with preexisting diabetes mellitus may not be applicable during pregnancy. A previous study had suggested a lower HbA1c cutoff of ≥5.9%. However, only 15 participants met the criteria of DIP diagnosed using OGTT.^10^^,^^11^ HbA1c in early pregnancy, before the conventional OGTT testing at 24 to 28 weeks of gestation, has the potential to identify women with possible preexisting diabetes mellitus and women at risk of hyperglycemic pregnancy complications, but the appropriate cutoff remains unclear. Therefore, we conducted this study to evaluate the diagnostic performance of various HbA1c levels and the optimal HbA1c cutoff to identify mothers with DIP diagnosed by the gold standard 75 g OGTT before 24 weeks of gestation. In addition, we compared the pregnancy and neonatal outcomes using the optimal HbA1c cutoff.

## Materials and Methods

This was a retrospective study conducted at Queen Mary Hospital (QMH), a university-affiliated hospital in Hong Kong, between 2004 and 2019. In QMH, all women attending antenatal visits were routinely screened for risk factors of GDM at their first booking visit, which included advanced maternal age (≥35 years old), obesity (a body mass index [BMI] of ≥ 25 kg/m^2^), polycystic ovarian syndrome, multiple pregnancy, polyhydramnios, glycosuria, personal history of unexplained stillbirth, macrosomia (fetal weight ≥4000 g) or GDM in previous pregnancy, and family history of diabetes mellitus. Women with any one of the aforementioned risk factors underwent an early 75 g OGTT before 24 weeks of gestation. HbA1c was not routinely checked at the time of early OGTT but was examined at the discretion of the attending obstetricians after the diagnosis of DIP or GDM. The terminology of hyperglycemia first detected using OGTT during pregnancy was classified as either DIP or GDM following the World Health Organization (WHO) recommendation.^3^ DIP was defined as either a fasting plasma glucose (FG) of ≥7.0 mmol/L and/or a 2-hour plasma glucose (2hG) of ≥11.1 mmol/L after OGTT.^3^

The WHO recommended the change in diagnostic criteria of GDM in 2013, which was later adopted in our hospital on July 1, 2015. Therefore, GDM was diagnosed as either an FG between 6.0 and 6.9mmol/L and/or a 2hG between 7.8 and 11.0 mmol/L after OGTT (before July 1, 2015) or an FG between 5.1 and 6.9 mmol/L and/or a 2hG between 8.5 and 11.0 mmol/L after OGTT (after July 1, 2015).^3^ Women with GDM or DIP received the same advice on lifestyle modification, self-monitoring of blood glucose, and dietitian referral. Hemoglobin pattern was performed in women with a mean corpuscular volume (MCV) of <82 fL. Suspected iron deficiency was defined as either an MCV of <82 fL with a normal hemoglobin pattern or a hemoglobin level of <11g/dL.^12^

Here, all women who underwent OGTT before 24 weeks of gestation because of the presence of any risk factor of GDM, met the diagnostic criteria of DIP or GDM (ie, early-onset GDM), and had a paired HbA1c measurement within 4 weeks of their early OGTT were included. Women were excluded if they had diabetes mellitus diagnosed before pregnancy, cirrhosis with hypersplenism, hemolytic anemia, or thalassemia major. Basic demographics and clinical details were recorded via the Clinical Data Analysis and Reporting System, which included the maternal age at their estimated date of confinement, parity, gestational age at birth, pregnancy complications, and neonatal outcomes. This study followed the Strengthening the Reporting of Observational Studies in Epidemiology reporting guideline. Ethical approval was obtained from the institutional review board of The University of Hong Kong and the Hospital Authority Hong Kong West Cluster, and informed consent was waived because of the retrospective design of this study.

### Laboratory measurements

Plasma specimens were collected in sodium fluoride-potassium oxalate tubes. Plasma glucose was measured using the hexokinase method on cobas 702 (Roche Diagnostics, Indianapolis, IN). HbA1c levels were measured using the D-100 Hemoglobin Testing System (Bio-Rad Laboratories, Hercules, CA). Hemoglobin species were separated using high-performance liquid chromatography and detected using absorbance at 415 nm. The results were traceable to the United Kingdom Prospective Diabetes Study and the Diabetes Chronic Complications Trial.

### Statistical analysis

Statistical analysis was performed using SPSS Statistics software (version 28.0; IBM Corporation, Armonk, NY). Normally distributed continuous data were presented as mean (standard deviation), whereas skewed continuous data were presented as median (interquartile range). Categorical data were presented as number (percentage). Comparisons of clinical characteristics and maternal and pregnancy outcomes between groups were performed using the independent *t* test for normally distributed continuous variables or the Mann-Whitney *U* test for skewed continuous variables. For categorical variables, comparisons were performed using the chi-square test, Fisher exact test, or likelihood ratio test, as appropriate. Of note, 2 sets of sensitivity, specificity, positive predictive value (PPV), and negative predictive value (NPV) and their respective 95% confidence intervals (CIs) at various HbA1c cutoffs were calculated in the (1) whole cohort and (2) after exclusion of women with possible hemoglobinopathy and iron deficiency anemia. Receiver operating characteristic (ROC) curves were plotted on SPSS and adapted on Microsoft PowerPoint. To investigate the associations between maternal HbA1c level and pregnancy outcomes, univariate and multivariate binary logistic regression analyses were performed to calculate the unadjusted odds ratio (OR) and adjusted OR (aOR). A 2-sided *P* value of <.05 was considered statistically significant.

## Results

There were 63,111 deliveries between 2004 and 2019 (45,949 before and 17,162 after July 1, 2015), of which 22,949 women underwent OGTT before 24 weeks of gestation (15,755 before and 7194 after July 1, 2015). A total of 157 (99 before and 58 after July 1, 2015) and 3210 (2436 before and 774 after July 1, 2015) women met the diagnostic criteria of DIP and early-onset GDM using OGTT, respectively. Only 346 participants had a paired HbA1c and OGTT measurement (82 with DIP and 264 with early-onset GDM) (Figure). All women did not have diabetes mellitus before pregnancy, cirrhosis with hypersplenism, hemolytic anemia, or thalassemia major. Moreover, 5 women (2 with DIP and 3 with early-onset GDM) had a recurrence in subsequent pregnancies, and their deliveries were included in the analysis. There were no missing data. The baseline demographic is shown in Table 1. The median HbA1c level was 5.4% (5.2%–5.8%), taken at a median of 17.0 gestational weeks (15.0–19.4). Among the 82 women with DIP, 22 had an HbA1c level of ≥6.5%, whereas 3 women in the early-onset GDM group had an HbA1c level of ≥6.5%. Early OGTT was performed at a median gestational week of 15.9 (13.4–17.8). Compared with women who had early-onset GDM, women with DIP had significantly higher BMI (23.5 vs 25.7 kg/m^2^, respectively; *P*<.001); were more likely to be multiparous (41.7% vs 59.8%, respectively; *P*=.004); received their early OGTT at higher gestational weeks (15.6 vs 16.4 weeks, respectively; *P*=.028); had higher FG, 2hG, and HbA1c values (4.4 vs 5.8 mmol/L, respectively; *P*<.001; 8.5 vs 12.4 mmol/ L, respectively; *P*<.001; 5.3% vs 5.9%, respectively; *P*<.001). There was no significant difference in maternal age, ethnicity, hemoglobin and MCV level at booking visit, gestational age of HbA1c testing, and proportion of multiple pregnancy and underlying hemoglobinopathy between women with and without DIP.

**Figure:**
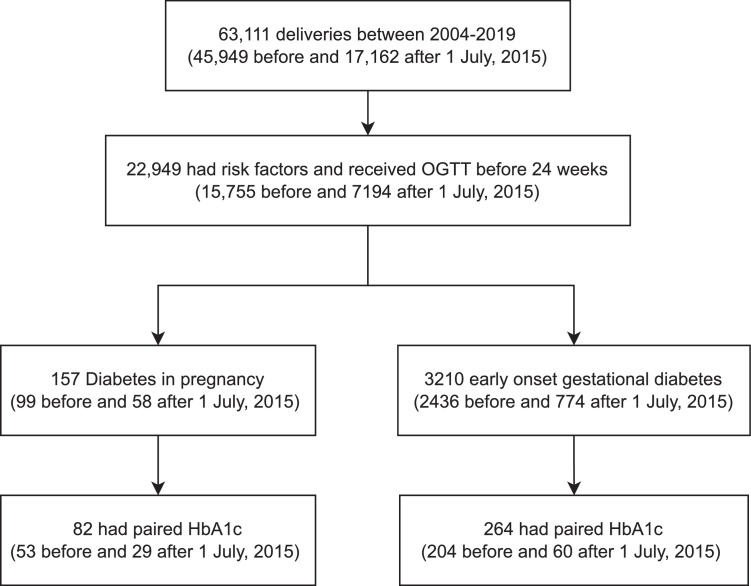
Flow diagram *OGTT*, oral glucose tolerance test.

Cheung. Hemoglobin A1c and diabetes mellitus in pregnancy. Am J Obstet Gynecol Glob Rep 2024.

**Table 1:** Basic demographics between women with DIP and women with GDM

Characteristics	Total (N=346)	GDM in pregnancy (n=264)	DIP (n=82)	*P* value
	n (%)	n (%)	n (%)	
Maternal age at estimated date of confinement (y)				.233
• Mean (SD)	36.3 (4.2)	36.2 (4.2)	36.8 (4.2)	
Body mass index (kg/m^2^)				<.001
• Median (IQR)	24.0 (21.5–27.0)	23.5 (21.3–26.5)	25.7 (23.0–27.8)	
Ethnicity				.548
• Chinese	306 (8.4)	235 (89.0)	71 (86.6)	
• Others	40 (11.6)	29 (11.0)	11 (13.4)	
Parity				.004
• Nulliparity	187 (54.0)	154 (58.3)	33 (40.2)	
Multiple pregnancy				.153
• Twin pregnancy	39 (11.3)	33 (12.5)	6 (7.3)	
• Triplet pregnancy	6 (1.7)	6 (2.3)	0 (0)	
Hemoglobin level at booking (g/dL)				.168
• Median (IQR)	12.5 (11.7–13.1)	12.4 (11.6–13.1)	12.6 (12.0–13.2)	
MCV level at booking (fL)				.722
• Median (IQR)	88.6 (85.5–91.6)	88.6 (85.6–91.7)	88.6 (83.8–91.5)	
Underlying hemoglobinopathy				.464
• Thalassemia	31 (9.0)	22 (8.3)	9 (11.0)	
Gestational age of OGTT (wk)				.028
• Median (IQR)	15.9 (13.4–17.8)	15.6 (13.1–17.6)	16.4 (14.8–18.5)	
Fasting glucose (mmol/L)				<.001
• Median (IQR)	4.5 (4.2–5.2)	4.4 (4.2–4.7)	5.8 (4.8–6.7)	
2-h glucose (mmol/L)				<.001
• Median (IQR)	9.0 (8.0–11.0)	8.5 (7.8–9.6)	12.4 (11.5–13.3)	
Gestational age of HbA1c (wk)				.183
• Median (IQR)	17.0 (15.0–19.4)	16.9 (14.8–19.3)	17.4 (15.4–19.6)	
HbA1c level (%)				<.001
• Median (IQR)	5.4 (5.2–5.8)	5.3 (5.1–5.6)	5.9 (5.5–6.5)	

*DIP*, diabetes in pregnancy; *GDM*, gestational diabetes mellitus; *HbA1c*, hemoglobin A1c; *IQR*, interquartile range; *MCV*, mean corpuscular volume *OGTT*, oral glucose tolerance test; *SD*, standard deviation.

Supplementary Table S1 shows the sensitivity, specificity, PPV, and NPV at different HbA1c cutoffs to identify DIP diagnosed using OGTT. The HbA1c cutoffs of 5.9% and 6.5% achieved a sensitivity of 52.4% and 26.8%, respectively. Supplementary Figure 1, A, shows the ROC curve (area under the curve [AUC] of 0.786), which suggested an optimal HbA1c cutoff of 5.7% to diagnose DIP, with a sensitivity of 64.6% (95% CI, 54.0%–74.4%), specificity of 81.1% (95% CI, 76.0%–85.5%), PPV of 51.5% (95% CI, 41.9%–61.0%), and NPV of 88.1% (95% CI, 83.6%–91.7%).

In a sensitivity analysis that excluded 68 subjects with thalassemia traits or suspected iron deficiency, the sensitivity, specificity, PPV, and NPV of using various levels of HbA1c as a cutoff to identify DIP as diagnosed using OGTT are shown in Supplementary Table S2. As suggested by the ROC curve (AUC of 0.781), the optimal HbA1c cutoff was 5.8% (Supplementary Figure 1, B), which achieved a sensitivity of 59.7% (95% CI, 47.8%–70.9%), specificity of 86.3% (95% CI, 81.2%–90.5%), PPV of 58.0% (95% CI, 46.2%–69.2%), and NPV of 87.1% (95% CI, 82.1%–91.2%).

Supplementary Table S3 shows the basic demographics of women stratified using the optimal HbA1c cutoff of 5.7%. Compared with women with an HbA1c level of <5.7%, women with an HbA1c level of ≥5.7% had a higher baseline BMI (23.3 vs 26.4 kg/m^2^, respectively), FG (4.4 vs 5.5 mmol/L, respectively), and 2hG levels (8.6 vs 11.1 mmol/L, respectively) (all *P*<.001). Moreover, there were significantly more women with an HbA1c level of ≥5.7% requiring insulin to optimize glycemic control than women with an HbA1c level of <5.7% (37.9% vs 6.6%, respectively; *P*<.001) (Table 2). Moreover, compared with the newborns of women with an HbA1c level of <5.7%, the newborns of women with an HbA1c level of ≥5.7% were significantly heavier (2775.0 vs 3127.5 g, respectively; *P*<.001), were more likely to be macrosomic (1.0% vs 7.4%, respectively; *P*<.001), and had shoulder dystocia (0.7% vs 3.7%, respectively; *P*=.049). There was no significant difference in other pregnancy and neonatal outcomes (Table 2). In multivariate logistic regression analysis, an HbA1c level of ≥5.7% was independently associated with an increased risk of maternal insulin use (aOR, 6.69; 95% CI, 3.44–12.99), macrosomia (aOR, 7.43; 95% CI, 1.90–29.00), and shoulder dystocia (aOR, 6.56; 95% CI, 1.161–37.032), after adjustments for maternal BMI at baseline (Table 3). The findings were similar when pregnancy and neonatal outcomes were compared between women with an HbA1c level of <5.7% and women with an HbA1c level of 5.7% to 6.4%. (Supplementary Tables S4–S6).

**Table 2:** Comparison of maternal and neonatal outcomes between mothers with an HbA1c level of <5.7% and mothers with an HbA1c level of ≥5.7%

Outcomes	Total (N=346)	HbA1c<5.7% (n=243)	HbA1c≥5.7% (n=103)	*P* value
	n (%) or median (IQR)	n (%) or median (IQR)	n (%) or median (IQR)	
Insulin for maternal glycemic control	55 (15.9)	16 (6.6)	39 (37.9)	<.001
Gestational hypertension	22 (6.4)	13 (5.3)	9 (8.7)	.237
Preeclampsia	19 (5.5)	10 (4.1)	9 (8.7)	.118
	N=370^a^	n=267^a^	n=103^a^	
Gestational age at delivery (wk)	38.0 (36.7–38.9)	38.0 (36.4–39.0)	38.1 (37.1–38.9)	.254
Preterm birth among single pregnancy (<37 wk)	46 (16.1)	29 (15.3)	17 (17.9)	.569
Mode of delivery				.403
• Natural spontaneous delivery	142 (38.4)	100 (37.5)	42 (40.8)	
• Forceps delivery	8 (2.2)	7 (2.6)	1 (1.0)	
• Vacuum extraction	18 (4.9)	13 (4.9)	5 (4.9)	
• Elective lower segment cesarean delivery	95 (25.7)	73 (27.3)	22 (21.4)	
• Emergency lower segment cesarean delivery	104 (28.1)	71 (26.6)	33 (32.0)	
• Emergency classical cesarean delivery	3 (0.8)	3 (1.1)	0 (0)	
	N=397	n=289	n=108	
Fetal outcome				.520
• Livebirth	369 (92.9)	266 (92.0)	193 (95.4)	
• Miscarriage	26 (6.5)	21 (7.3)	5 (4.6)	
• Stillbirth	1 (0.3)	1 (0.3)	(0.0)	
• Neonatal death	1 (0.3)	1 (0.3)	0 (0.0)	
Birthweight (g)				
• Median (IQR)	2905.0 (2275.0–3325.0)	2775.0 (2137.5–3167.5)	3127.5 (2740.0–3481.3)	<.001
• >4000 g	11 (2.8)	3 (1.0)	8 (7.4)	<.001
Apgar score at 1 min				.994
• Median (IQR)	9.0 (9.0–9.0)	9.0 (9.0–9.0)	9.0 (9.0–9.0)	
Apgar score at 5 min				.786
• Median (IQR)	10.0 (10.0–10.0)	10.0 (10.0–10.0)	10.0 (10.0–10.0)	
Shoulder dystocia	6 (1.5)	2 (0.7)	4 (3.7)	.049
Congenital anomaly	2 (0.5)	2 (0.7)	0 (0)	1.000
Respiratory distress syndrome	3 (0.8)	3 (1.0)	0 (0)	.566
Necrotizing enterocolitis	1 (0.3)	1 (0.3)	0 (0)	1.000
Intraventricular hemorrhage	3 (0.8)	2 (0.7)	1 (0.9)	1.000
Neonatal hypoglycemia	23 (5.8)	13 (4.5)	10 (9.3)	.071
NICU admission for >24 h	8 (2.0)	5 (1.7)	3 (2.8)	.453

*HbA1c*, hemoglobin A1c; *IQR*, interquartile range; *NICU*, neonatal intensive care unit.

a Miscarriages and stillbirth were excluded.

**Table 3:** ORs showing the association between maternal HbA1c level of ≥5.7% and pregnancy outcomes

Outcomes	Unadjusted OR (95% CI)	*P* value	Adjusted OR (95% CI)^a^	*P* value
Gestational hypertension	1.694 (0.700–4.097)	.242	1.517 (0.599–3.843)	.380
Preeclampsia	2.231 (0.879–5.665)	.091	1.957 (0.734–5.220)	.180
Insulin use for maternal glycemic control	8.646 (4.538–16.472)	<.001	6.685 (3.440–12.991)	<.001
Preterm birth among singleton pregnancy (<37 wk)	1.210 (0.627–2.334)	.570	1.211 (0.622–2.358)	.574
Birthweight>4000 g	7.627 (1.984–29.311)	.003	7.433 (1.904–29.007)	.004
Shoulder dystocia	5.519 (0.996–30.581)	.051	6.557 (1.161–37.032)	.033
Intraventricular hemorrhage	1.341 (0.120–14.942)	.811	1.254 (0.109–14.478)	.856
Neonatal hypoglycemia	2.166 (0.920–5.099)	.077	2.035 (0.853–4.854)	.109
NICU admission for >24 h	1.623 (0.381–6.910)	.512	1.820 (0.420–7.882)	.423

*CI*, confidence interval; *HbA1c*, hemoglobin A1c; *NICU*, neonatal intensive care unit; *OR*, odds ratio.

a Adjusted for maternal body mass index.

## Discussion

### Principal findings

The current study demonstrated that the optimal HbA1c cutoff to detect DIP diagnosed by OGTT before 24 weeks of gestation was 5.7%. Using the previous recommended cutoff of 6.5% or the optimal cutoff derived in our study could not reliably identify DIP diagnosed by OGTT owing to the low sensitivity. However, our study found that women with an HbA1c level of ≥5.7% before 24 weeks of gestation had significantly higher risks of pregnancy and neonatal complications, including the need for insulin to optimize maternal glycemic control and the incidences of macrosomia and shoulder dystocia.

### Clinical and research implications

The growing global prevalence of T2D among women of reproductive age could mean a higher proportion of women with unrecognized preexisting T2D during pregnancy. Between 1998 and 2013, pregnancy complicated by T2D increased significantly by 90% in Scotland.^2^ Similarly, in Hong Kong, the incidence of T2D among women aged 20 to 40 years increased significantly between 2002 and 2015, from 45.0 to 62.1 per 100,000 person-years.^13^ Earlier detection and intervention could be made possible by incorporating HbA1c into the routine antenatal screening program in early pregnancy and, thus, reduce the associated increased risk of pregnancy complications with unrecognized preexisting diabetes mellitus.^2^ Interpretation of HbA1c should be adjusted for physiological changes during pregnancy, and gestational-specific HbA1c was proposed to manage hyperglycemia in pregnancy.^14^ Compared with age-matched nonpregnant women, HbA1c levels of women without hyperglycemia reduced in early pregnancy. The upper normal limit of HbA1c in early pregnancy was 5.7%, which was 0.6% less than the upper limit in nonpregnant women.^15^ In addition, the FG level decreased in early pregnancy, in particular during 6 to 10 weeks of gestation, and the level of reduction ranged from 0.11 to 0.31 mmol/L.^16^ Physiological metabolic and hormonal changes and increased fetal glucose use could account for the reduction of glucose levels during pregnancy. Moreover, the shortened life span of the erythrocyte during pregnancy reduces the exposure to average glucose concentration, which ultimately affects glycosylation and brings down the HbA1c level.^15^ Hughes et al^10^ prospectively evaluated the accuracy of HbA1c, taken at a median gestation of 47 days in unselected pregnant women, to identify DIP diagnosed using OGTT. They recommended a cutoff of 5.9% to screen for DIP, with 100% sensitivity, 97.4% specificity, 18.8% PPV, and 100% NPV. Using 6.5% as a cutoff missed 46.7% (7/15) of women with DIP detected using OGTT. Moreover, pregnant women with an HbA1c level between 5.9% and 6.4% had a higher risk of congenital anomaly, preeclampsia, shoulder dystocia, and perinatal death, compared with those with an HbA1c level of <5.9%. However, lower uptake of early OGTT before 20 weeks of gestation (especially in women with an HbA1c level of <5.9%), only 15 subjects fulfilled the OGTT criteria for DIP, and no adjustment for iron deficiency and hemoglobinopathy were potential limitations for its wider clinical application.^11^ Iron deficiency and underlying hemoglobinopathy could influence HbA1c levels during pregnancy. The exact mechanism of iron deficiency leading to an elevated HbA1c level remained unclear.^17^ Iron deficiency is more common during pregnancy because of increased iron requirement to support red blood cell mass expansion and fetal need.^18^ Even in the first trimester of pregnancy, iron deficiency could occur in 42% of nonanemic pregnant women. In hemoglobinopathies, shortened red blood cells’ life span and associated iron overload could lead to a lower level of HbA1c.^19^^,^^20^ Nonetheless, we found that the optimal HbA1c cutoff was similar after the exclusion of women with hemoglobinopathy and suspected iron deficiency.

The International Association of Diabetes and Pregnancy Study Groups recommended an HbA1c cutoff of 6.5% to identify DIP early in pregnancy.^7^^,^^8^ However, our study suggested that no single HbA1c cutoff was ideal as a screening tool. Using either HbA1c cutoff of 5.7%, 5.9%, or 6.5% could miss 35.4%, 47.6%, or 73.2% of women with DIP diagnosed using OGTT in our cohort, respectively. In addition, the PPV of 51.5% signified not all women with an HbA1c level of ≥5.7% would meet the diagnostic criteria of DIP using OGTT.

Although HbA1c could not replace OGTT to detect DIP, it may be useful to identify women with a milder degree of hyperglycemia who are at a higher risk of developing adverse pregnancy outcomes. In our cohort, the difference in pregnancy complications between women with an HbA1c level of <5.7% and women with an HbA1c level of ≥5.7% suggested that the cutoffs could be used in early pregnancy to recognize women at a higher risk of developing complications because of hyperglycemia. Interestingly, the HbA1c level of 5.7% (and below 6.5%) was used to define prediabetes in nonpregnant individuals^5^ and had been examined for its implication on pregnancy outcomes.^21^^,^^22^ Although these studies revealed that women with a first-trimester HbA1c level of 5.7% to 6.4% had an increased risk of GDM at 24 to 28 weeks of gestation and a higher need for pharmacologic treatment, no adverse pregnancy complication was found when compared with women with an HbA1c level of <5.7%. In these studies, OGTTs were performed in women with prediabetes (HbA1c level of 5.7% of 6.4%) to triage subsequent management. Subsequently, women diagnosed with early-onset GDM using OGTT received GDM interventions, which may reduce pregnancy complications and mask the adverse effects of hyperglycemia. The comparable pregnancy outcomes between women with an HbA1c level of < 5.7% and women with an HbA1c level of ≥ 5.7% demonstrated the importance of early treatment in women with prediabetes who failed an OGTT, as further highlighted by the persistent increased risk of complications in women with an HbA1c level between 5.7% and 6.4% in our cohort.

Currently, there is no consensus on the diagnostic criteria to screen for early-onset GDM before 24 to 28 weeks of gestation based on OGTT values.^23^^,^^24^ Using the World Health Organization's criteria could identify 21.8% of women with risk factors for early-onset GDM before 20 weeks of gestation. Although earlier identification and treatment could reduce the risk of neonatal respiratory distress, it may increase the risk of a small-for-gestational-age fetus in women with a lower glycemic range. Furthermore, without any intervention, one-third of women recovered from early-onset GDM when they had repeated testing at 24 to 28 weeks of gestation.^25^ Until normative data of OGTT values in early pregnancy and the risk of pregnancy complications from early gestational hyperglycemia are available, HbA1c may be a feasible alternative as routine antenatal screening in early pregnancy to detect women at a higher risk of pregnancy complications because of hyperglycemia.

### Strengths and limitations

The strengths of the study were that we accounted for the effect of iron deficiency and hemoglobinopathy on the HbA1c level and had a larger sample size of women with DIP in this study than in the previous study. There were several limitations in our study. First, this was a retrospective study, and there was selection bias as HbA1c was not examined in some women with DIP or GDM and all women without diabetes mellitus. The levels of HbA1c could differ among these women and affect our findings and interpretation. Nonetheless, our results echoed other reports that an early pregnant HbA1c level of 5.7% to 6.4% in women without diabetes mellitus was associated with an increased risk of adverse pregnancy outcomes.^26^ Second, HbA1c testing was not performed at the same time but within 4 weeks of OGTT. The effect of this slight delay of HbA1c testing should be minimal as HbA1c reflects the average glycemic control over the past 8 to 12 weeks, and the median time interval between OGTT and HbA1c in our cohort was only 1 to 2 weeks. Third, we only included women with risk factors of developing GDM and not every woman had HbA1c testing. The cost-effectiveness of routine HbA1c screening for maternal hyperglycemia in low-risk and high-risk women would require further evaluation. Finally, most women had HbA1c measured during the second trimester of pregnancy when its value was physiologically at its nadir. However, a second-trimester HbA1c still enabled us to identify women at higher risk of pregnancy complications. With these limitations, we believe that the prospective evaluation of universal HbA1c assessment in early pregnancy to identify women at risk of hyperglycemic complications is necessary.

### Conclusion

The optimal HbA1c cutoff to detect DIP diagnosed using OGTT before 24 weeks of gestation was 5.7%, but this cutoff could not reliably identify DIP owing to the low sensitivity. However, an early HbA1c level of ≥5.7% indicated increased risks of using maternal insulin, macrosomia, and shoulder dystocia.

## CRediT authorship contribution statement

**Ka Wang Cheung:** Conceptualization, Data curation, Investigation, Methodology, Supervision, Writing – original draft, Writing – review & editing. **Tiffany Sin-Tung Au:** Conceptualization, Data curation, Formal analysis, Investigation, Methodology, Writing – original draft, Writing – review & editing. **Chi-Ho Lee:** Supervision, Writing – review & editing. **Vivian Wai Yan Ng:** Supervision, Writing – review & editing. **Felix Chi-Kin Wong:** Data curation, Methodology, Supervision, Writing – review & editing. **Wing-Sun Chow:** Supervision, Writing – review & editing. **Pui Wah Hui:** Supervision, Writing – review & editing. **Mimi Tin Yan Seto:** Conceptualization, Project administration, Supervision, Writing – review & editing.

## Appendix Supplementary materials

Image, application 1

## Footnotes

Supplementary material associated with this article can be found in the online version at doi:10.1016/j.xagr.2024.100315.
