# Supplementary material for: Hemoglobin A1c in early pregnancy to identify preexisting diabetes mellitus and women at risk of hyperglycemic pregnancy complications
Source: AJOG Glob Rep. 2024 Jan 19;4(1):100315. doi: 10.1016/j.xagr.2024.100315 (PMC10867763; doi:10.1016/j.xagr.2024.100315)
Supplement: Supplementary file 1 [file mmc1.docx]

| **Supplementary table 1. Sensitivity, specificity, positive predictive value and negative predictive value at different HbA1c cut-offs for identification of DM in pregnancy** | | | | |
| --- | --- | --- | --- | --- |
| **HbA1c cut-off** | **Sensitivity**  **% (95% CI)** | **Specificity**  **% (95% CI)** | **Positive predictive value**  **% (95% CI)** | **Negative predictive value**  **% (95% CI)** |
| **HbA1c ≥ 4.5%** | 100.0 (100.0-100.0) | 1.5 (0.5-3.5) | 24.0 (19.7-28.7) | 100.0 (100.0-100.0) |
| **HbA1c ≥ 4.6%** | 100.0 (100.0-100.0) | 3.4 (1.7-6.1) | 24.3 (20.0-29.1) | 100.0 (100.0-100.0) |
| **HbA1c ≥ 4.7%** | 100.0 (100.0-100.0) | 4.5 (2.5-7.5) | 24.6 (20.1-29.3) | 100.0 (100.0-100.0) |
| **HbA1c ≥ 4.8%** | 98.8 (94.7-99.9) | 6.4 (3.9-9.8) | 24.7 (20.2-29.5) | 94.4 (77.7-99.7) |
| **HbA1c ≥ 4.9%** | 96.3 (90.8-99.1) | 8.3 (5.4-12.1) | 24.6 (20.1-29.5) | 88.0 (71.8-96.9) |
| **HbA1c ≥ 5.0%** | 96.3 (90.8-99.1) | 14.0 (10.2-18.5) | 25.8 (21.1-30.9) | 92.5 (81.7-98.1) |
| **HbA1c ≥ 5.1%** | 93.9 (87.4-97.8) | 20.5 (15.9-25.6) | 26.8 (21.9-32.1) | 91.5 (82.7-96.9) |
| **HbA1c ≥ 5.2%** | 93.9 (87.4-97.8) | 25.4 (20.4-30.8) | 28.1 (23.0-33.6) | 93.1 (85.7-97.5) |
| **HbA1c ≥ 5.3%** | 87.8 (79.6-93.7) | 37.9 (32.2-43.8) | 30.5 (24.9-36.6) | 90.9 (84.6-95.3) |
| **HbA1c ≥ 5.4%** | 84.1 (75.2-90.9) | 51.1 (45.1-57.1) | 34.8 (28.4-41.6) | 91.2 (86.0-95.1) |
| **HbA1c ≥ 5.5%** | 78.0 (68.3-86.1) | 61.4 (55.4-67.1) | 38.6 (31.4-46.1) | 90.0 (85.1-93.8) |
| **HbA1c ≥ 5.6%** | 68.3 (57.8-77.7) | 71.2 (65.6-76.5) | 42.4 (34.2-50.9) | 87.9 (83.0-91.8) |
| **HbA1c ≥ 5.7%** | 64.6 (54.0-74.4) | 81.1 (76.0-85.5) | 51.5 (41.9-61.0) | 88.1 (83.6-91.7) |
| **HbA1c ≥ 5.8%** | 59.8 (49.0-69.9) | 85.6 (81.0-89.5) | 56.3 (45.8-66.4) | 87.3 (82.8-90.9) |
| **HbA1c ≥ 5.9%** | 52.4 (41.7-63.0) | 90.2 (86.2-93.4) | 62.3 (50.6-73.2) | 85.9 (81.5-89.7) |
| **HbA1c ≥ 6.0%** | 47.6 (37.0-58.3) | 92.8 (89.3-95.5) | 67.2 (54.6-78.4) | 85.1 (80.7-88.9) |
| **HbA1c ≥ 6.1%** | 42.7 (32.3-53.5) | 96.2 (93.5-98.1) | 77.8 (64.3-88.2) | 84.4 (80.0-88.2) |
| **HbA1c ≥ 6.2%** | 35.4 (25.6-46.0) | 97.0 (94.4-98.6) | 78.4 (63.5-89.5) | 82.8 (78.4-86.8) |
| **HbA1c ≥ 6.3%** | 32.9 (23.4-43.5) | 98.1 (96.0-99.3) | 84.4 (69.4-94.1) | 82.5 (78.0-86.4) |
| **HbA1c ≥ 6.4%** | 29.3 (20.2-39.7) | 98.5 (96.5-99.5) | 85.7 (69.9-95.3) | 81.8 (77.3-85.7) |
| **HbA1c ≥ 6.5%** | 26.8 (18.1-37.0) | 98.9 (97.1-99.7) | 88.0 (71.8-96.9) | 81.3 (76.8-85.3) |
| **HbA1c ≥ 6.6%** | 22.0 (13.9-31.7) | 98.9 (97.1-99.7) | 85.7 (67.0-96.2) | 80.3 (75.8-84.4) |
| **HbA1c ≥ 6.7%** | 18.3 (11.0-27.6) | 98.9 (97.1-99.7) | 83.3 (62.3-95.6) | 79.6 (75.0-83.7) |
| **HbA1c ≥ 6.8%** | 17.1 (10.0-26.2) | 98.9 (97.1-99.7) | 82.4 (60.4-95.3) | 79.3 (74.7-83.5) |
| **HbA1c ≥ 6.9%** | 14.6 (8.1-23.3) | 99.2 (97.7-99.9) | 85.7 (62.1-97.5) | 78.9 (74.3-83.1) |
| **HbA1c ≥ 7.0%** | 13.4 (7.2-21.9) | 99.6 (98.3-100.0) | 91.7 (68.1-99.5) | 78.7 (74.1-82.9) |
| **HbA1c ≥ 7.5%** | 6.1 (2.2-12.6) | 99.6 (98.3-100.0) | 83.3 (44.6-99.0) | 77.4 (72.7-81.6) |

| **Supplementary table 2. Sensitivity, specificity, positive predictive value and negative predictive value at different HbA1c cut-offs for identification of diabetes in pregnancy after exclusion of subjects with haemoglobinopathy, haemoglobin level < 11g/dL or MCV < 82 fL.** | | | | |
| --- | --- | --- | --- | --- |
| **HbA1c cut-off** | **Sensitivity**  **% (95% CI)** | **Specificity**  **% (95% CI)** | **Positive predictive value**  **% (95% CI)** | **Negative predictive value**  **% (95% CI)** |
| **HbA1c ≥ 4.5%** | 100.0 (100.0-100.0) | 1.4 (0.4-3.6) | 24.4 (19.5-29.7) | 100.0 (100.0-100.0) |
| **HbA1c ≥ 4.6%** | 100.0 (100.0-100.0) | 2.8 (1.1-5.7) | 24.6 (19.8-30.0) | 100.0 (100.0-100.0) |
| **HbA1c ≥ 4.7%** | 100.0 (100.0-100.0) | 4.3 (2.1-7.6) | 24.9 (20.0-30.3) | 100.0 (100.0-100.0) |
| **HbA1c ≥ 4.8%** | 98.5 (93.6-99.9) | 6.6 (3.8-10.5) | 25.1 (20.1-30.6) | 93.3 (73.8-99.6) |
| **HbA1c ≥ 4.9%** | 95.5 (88.8-98.9) | 8.1 (4.9-12.2) | 24.8 (19.8-30.3) | 85.0 (65.6-96.0) |
| **HbA1c ≥ 5.0%** | 95.5 (88.8-98.9) | 12.3 (8.3-17.2) | 25.7 (20.5-31.4) | 89.7 (75.3-97.3) |
| **HbA1c ≥ 5.1%** | 94.0 (86.7-98.1) | 19.0 (14.1-24.6) | 26.9 (21.5-32.8) | 90.9 (80.1-97.1) |
| **HbA1c ≥ 5.2%** | 94.0 (86.7-98.1) | 24.2 (18.7-30.2) | 28.3 (22.6-34.4) | 92.7 (83.9-97.7) |
| **HbA1c ≥ 5.3%** | 86.6 (77.1-93.3) | 38.4 (32.0-45.1) | 30.9 (24.5-37.7) | 90.0 (82.7-95.1) |
| **HbA1c ≥ 5.4%** | 83.6 (73.5-91.1) | 50.7 (44.0-57.4) | 35.0 (27.9-42.6) | 90.7 (84.6-95.0) |
| **HbA1c ≥ 5.5%** | 79.1 (68.4-87.6) | 60.7 (54.0-67.1) | 39.0 (31.0-47.3) | 90.1 (84.5-94.3) |
| **HbA1c ≥ 5.6%** | 68.7 (57.0-78.9) | 70.6 (64.2-76.5) | 42.6 (33.5-52.0) | 87.6 (82.1-92.0) |
| **HbA1c ≥ 5.7%** | 64.2 (52.3-75.0) | 81.5 (75.9-86.4) | 52.4 (41.7-63.0) | 87.8 (82.7-91.8) |
| **HbA1c ≥ 5.8%** | 59.7 (47.8-70.9) | 86.3 (81.2-90.5) | 58.0 (46.2-69.2) | 87.1 (82.1-91.2) |
| **HbA1c ≥ 5.9%** | 52.2 (40.4-63.9) | 89.6 (85.0-93.2) | 61.4 (48.5-73.3) | 85.5 (80.5-89.7) |
| **HbA1c ≥ 6.0%** | 46.3 (34.6-58.2) | 92.4 (88.3-95.5) | 66.0 (51.8-78.4) | 84.4 (79.4-88.7) |
| **HbA1c ≥ 6.1%** | 41.8 (30.5-53.7) | 95.7 (92.4-97.9) | 75.7 (60.4-87.5) | 83.8 (78.8-88.1) |
| **HbA1c ≥ 6.2%** | 35.8 (25.0-47.7) | 96.2 (93.1-98.2) | 75.0 (58.4-87.7) | 82.5 (77.5-86.9) |
| **HbA1c ≥ 6.3%** | 32.8 (22.4-44.6) | 97.6 (95.0-99.1) | 81.5 (64.3-92.9) | 82.1 (77.0-86.5) |
| **HbA1c ≥ 6.4%** | 29.9 (19.8-41.4) | 98.1 (95.7-99.4) | 83.3 (65.4-94.5) | 81.5 (76.4-85.9) |
| **HbA1c ≥ 6.5%** | 26.9 (17.3-38.2) | 98.6 (96.4-99.6) | 85.7 (67.0-96.2) | 80.9 (75.8-85.4) |
| **HbA1c ≥ 6.6%** | 22.4 (13.6-33.3) | 98.6 (96.4-99.6) | 83.3 (62.3-95.6) | 80.0 (74.9-84.6) |
| **HbA1c ≥ 6.7%** | 17.9 (10.0-28.2) | 98.6 (96.4-99.6) | 80.0 (56.0-94.6) | 79.1 (73.9-83.7) |
| **HbA1c ≥ 6.8%** | 16.4 (8.9-26.5) | 98.6 (96.4-99.6) | 78.6 (53.4-94.2) | 78.8 (73.6-83.4) |
| **HbA1c ≥ 6.9%** | 13.4 (6.7-22.9) | 99.1 (97.1-99.8) | 81.8 (53.7-96.7) | 78.3 (73.1-82.9) |
| **HbA1c ≥ 7.0%** | 11.9 (5.6-21.1) | 99.5 (97.9-100.0) | 88.9 (59.5-99.3) | 78.1 (72.9-82.7) |
| **HbA1c ≥ 7.5%** | 4.5 (1.1-11.2) | 99.5 (97.9-100.0) | 75.0 (27.8-98.4) | 76.6 (71.4-81.4) |

| **Supplementary table 3. Basic demographics of subjects using HbA1c of 5.7% as cut-off** | | | | |
| --- | --- | --- | --- | --- |
|  | **Total**  **n = 346, n (%)** | **HbA1c < 5.7%**  **n = 243, n (%)** | **HbA1c ≥ 5.7%**  **n = 103, n (%)** | **p-value** |
| **Maternal age at estimated date of confinement, years** |  |  |  | 0.638 |
| Mean (SD) | 36.3 (4.2) | 36.2 (3.9) | 36.5 (4.9) |  |
| **Body mass index, kg/m^2^** |  |  |  | < 0.001 |
| Median (IQR) | 24.0 (21.5-27.0) | 23.3 (21.0-26.0) | 26.4 (23.2-29.3) |  |
| **Ethnicity** |  |  |  | 0.132 |
| Chinese | 306 (88.4) | 219 (90.1) | 87 (84.5) |  |
| Others | 40 (11.6) | 24 (9.9) | 16 (15.5) |  |
| **Parity** |  |  |  | 0.181 |
| Nulliparity | 187 (54.0) | 137 (56.4) | 50 (48.5) |  |
| **Multiple pregnancy** |  |  |  | 0.003 |
| Twin pregnancy | 39 (11.3) | 34 (14.0) | 5 (4.9) |  |
| Triplet pregnancy | 6 (1.7) | 6 (2.5) | 0 (0.0) |  |
| **Hemoglobin level at booking, g/dL** |  |  |  | 0.392 |
| Median (IQR) | 12.5 (11.7-13.1) | 12.4 (11.7-13.1) | 12.6 (11.7-13.2) |  |
| **MCV level at booking, fL** |  |  |  | 0.062 |
| Median (IQR) | 88.6 (85.5-91.6) | 89.1 (86.1-91.5) | 87.3 (83.4-91.7) |  |
| **Underlying hemoglobinopathy** |  |  |  | 0.120 |
| Thalassemia | 31 (9.0) | 18 (7.4) | 13 (12.6) |  |
| **Gestational age of OGTT, weeks** |  |  |  | 0.420 |
| Median (IQR) | 15.9 (13.4-17.8) | 15.9 (13.4-18.0) | 15.7 (12.7-17.6) |  |
| **Fasting glucose, mmol/L** |  |  |  | < 0.001 |
| Median (IQR) | 4.5 (4.2-5.2) | 4.4 (4.2-4.7) | 5.5 (4.6-6.5) |  |
| **2 hours glucose, mmol/L** |  |  |  | < 0.001 |
| Median (IQR) | 9.0 (8.0-11.0) | 8.6 (7.8-9.8) | 11.1 (9.0-12.5) |  |
| **Gestational age of HbA1c, weeks** |  |  |  | 0.176 |
| Median (IQR) | 17.0 (15.0-19.4) | 17.1 (15.0-19.4) | 16.7 (14.4-18.9) |  |
| **HbA1c level, %** |  |  |  | < 0.001 |
| Median (IQR) | 5.4 (5.2-5.8) | 5.3 (5.1-5.5) | 6.0 (5.8-6.4) |  |

| **Supplementary table 4. Basic demographics of subjects between women with HbA1c < 5.7 and HbA1c 5.7-6.4%** | | | | |
| --- | --- | --- | --- | --- |
|  | **Total**  **n = 321, n (%)** | **HbA1c < 5.7**  **n = 243, n (%)** | **HbA1c 5.7-6.4%**  **n = 78, n (%)** | **p-value** |
| **Maternal age at estimated date of confinement, years** |  |  |  | 0.177 |
| Mean (SD) | 36.4 (4.1) | 36.2 (3.9) | 36.7 (4.7) |  |
| **Body mass index, kg/m^2^** |  |  |  | < 0.001 |
| Median (IQR) | 23.7 (21.3-26.8) | 23.3 (21.0-26.0) | 26.2 (22.8-28.6) |  |
| **Ethnicity** |  |  |  | 0.462 |
| Chinese | 287 (89.4) | 219 (90.1) | 68 (87.2) |  |
| Others | 34 (10.6) | 24 (9.9) | 10 (12.8) |  |
| **Parity** |  |  |  | 0.325 |
| Nulliparity | 176 (54.8) | 137 (56.4) | 39 (50.0) |  |
| **Multiple pregnancy** |  |  |  | 0.027 |
| Twin pregnancy | 39 (12.1) | 34 (14.0) | 5 (6.4) |  |
| Triplet pregnancy | 6 (1.9) | 6 (2.5) | 0 (0.0) |  |
| **Hemoglobin level at booking, g/dL** |  |  |  | 0.910 |
| Median (IQR) | 12.4 (11.7-13.1) | 12.4 (11.7-13.1) | 12.5 (11.6-13.1) |  |
| **MCV level at booking, fL** |  |  |  | 0.141 |
| Median (IQR) | 88.8 (85.7-91.6) | 89.1 (86.1-91.5) | 87.9 (83.4-91.7) |  |
| **Underlying hemoglobinopathy** |  |  |  | 0.140 |
| Thalassemia | 28 (8.7) | 18 (7.4) | 10 (12.8) |  |
| **Gestational age of OGTT, weeks** |  |  |  | 0.399 |
| Median (IQR) | 15.9 (13.4-17.8) | 15.9 (13.4-18.0) | 15.6 (12.7-17.5) |  |
| **Fasting glucose, mmol/L** |  |  |  | < 0.001 |
| Median (IQR) | 4.5 (4.2-5.0) | 4.4 (4.2-4.7) | 5.0 (4.4-6.0) |  |
| **2 hours glucose, mmol/L** |  |  |  | < 0.001 |
| Median (IQR) | 8.9 (7.9-10.4) | 8.6 (7.8-9.8) | 10.4 (8.7-11.8) |  |
| **Gestational age of HbA1c, weeks** |  |  |  | 0.265 |
| Median (IQR) | 17.0 (15.0-19.4) | 17.1 (15.0-19.4) | 16.9 (14.9-18.8) |  |
| **HbA1c level, %** |  |  |  | < 0.001 |
| Median (IQR) | 5.4 (5.2-5.6) | 5.3 (5.1-5.5) | 5.9 (5.8-6.1) |  |

| **Supplementary table 5. Comparison of maternal and neonatal outcomes between mothers with HbA1c < 5.7% and mothers with HbA1c 5.7-6.4%** | | | | |
| --- | --- | --- | --- | --- |
|  | **Total**  **n = 321, n (%) / median (IQR)** | **HbA1c < 5.7%**  **n = 243, n (%) / median (IQR)** | **HbA1c 5.7-6.4%**  **n = 78, n (%) / median (IQR)** | **p-value** |
| **Insulin for maternal glycemic control** | 34 (10.6) | 16 (6.6) | 18 (23.1) | < 0.001 |
| **Gestational hypertension** | 19 (5.9) | 13 (5.3) | 6 (7.7) | 0.419 |
| **Pre-eclampsia** | 18 (5.6) | 10 (4.1) | 8 (10.3) | 0.050 |
|  | **n = 346**  **n (%) / median (IQR)*** | **n = 267**  **n (%) / median (IQR)*** | **n = 79**  **n (%) / median (IQR)*** |  |
| **Gestational age at Delivery, weeks** | 38.0 (36.6-38.9) | 38.0 (36.4-39.0) | 38.0 (37.1-38.9) | 0.507 |
| **Preterm Birth among single pregnancy (< 37 weeks)** | 44 (16.9) | 29 (15.3) | 15 (21.1) | 0.260 |
| **Mode of Delivery** |  |  |  | 0.526 |
| Natural Spontaneous Delivery | 134 (38.7) | 100 (37.5) | 34 (43.0) |  |
| Forceps Delivery | 8 (2.3) | 7 (2.6) | 1 (1.3) |  |
| Vacuum Extraction | 17 (4.9) | 13 (4.9) | 4 (5.1) |  |
| Elective Lower Segment Caesarean Section | 89 (25.7) | 73 (27.3) | 16 (20.3) |  |
| Emergency Lower Segment Caesarean Section | 95 (27.5) | 71 (26.6) | 24 (30.4) |  |
| Emergency Classical Caesarean Section | 3 (0.9) | 3 (1.1) | 0 (0.0) |  |
|  | **n = 372**  **n (%) / median (IQR)** | **n = 289**  **n (%) / median (IQR)** | **n = 83**  **n (%) / median (IQR)** |  |
| **Fetal Outcome** |  |  |  | 0.637 |
| Livebirth | 345 (92.7) | 266 (92.0) | 79 (95.2) |  |
| Miscarriage | 25 (6.7) | 21 (7.3) | 4 (4.8) |  |
| Stillbirth | 1 (0.3) | 1 (0.3) | 0 (0.0) |  |
| Neonatal Death | 1 (0.3) | 1 (0.3) | 0 (0.0) |  |
| **Birth weight, g** |  |  |  |  |
| Median (IQR) | 2895.0 (2261.3-3316.3) | 2775.0 (2137.5-3167.5) | 3155.0 (2680.0-3570.0) | < 0.001 |
| > 4000g | 10 (2.7) | 3 (1.0) | 7 (8.4) | < 0.001 |
| **Apgar score at 1 min** |  |  |  | 0.818 |
| Median (IQR) | 9.0 (9.0-9.0) | 9.0 (9.0-9.0) | 9.0 (9.0-9.0) |  |
| **Apgar score at 5 mins** |  |  |  | 0.656 |
| Median (IQR) | 10.0 (10.0-10.0) | 10.0 (10.0-10.0) | 10.0 (10.0-10.0) |  |
| **Shoulder Dystocia** | 5 (1.3) | 2 (0.7) | 3 (3.6) | 0.076 |
| **Congenital Anomaly** | 2 (0.5) | 2 (0.7) | 0 (0.0) | 1.000 |
| **Respiratory Distress Syndrome** | 3 (0.8) | 3 (1.0) | 0 (0.0) | 1.000 |
| **Necrotizing Enterocolitis** | 1 (0.3) | 1 (0.3) | 0 (0.0) | 1.000 |
| **Intraventricular Hemorrhage** | 3 (0.8) | 2 (0.7) | 1 (1.2) | 0.532 |
| **Neonatal Hypoglycemia** | 17 (4.6) | 13 (4.5) | 4 (4.8) | 1.000 |
| **NICU Admission for > 24 hours** | 8 (2.2) | 5 (1.7) | 3 (3.6) | 0.384 |

*Miscarriages and stillbirth were excluded

| **Supplementary table 6. Odds ratios showing the association between maternal HbA1c 5.7-6.4% and pregnancy outcomes.** | | | | |
| --- | --- | --- | --- | --- |
|  | **Unadjusted Odds Ratio (95% CI)** | **p-value** | **Adjusted Odds Ratio (95% CI)*** | **p-value** |
| Gestational hypertension | 1.474 (0.541-4.019) | 0.448 | 1.242 (0.440-3.512) | 0.682 |
| Pre-eclampsia | 2.663 (1.012-7.006) | 0.047 | 2.455 (0.900-6.699) | 0.080 |
| Insulin use for maternal glycemic control | 4.256 (2.049-8.841) | < 0.001 | 3.361 (1.578-7.161) | 0.002 |
| Preterm Birth among singleton pregnancy (< 37 weeks) | 1.487 (0.743-2.975) | 0.262 | 1.472 (0.733-2.955) | 0.277 |
| Birth weight > 4000g | 8.781 (2.218-34.761) | 0.002 | 8.468 (2.126-33.727) | 0.002 |
| Shoulder dystocia | 5.381 (0.884-32.759) | 0.068 | 5.974 (0.971-36.769) | 0.054 |
| Intraventricular hemorrhage | 1.750 (0.157-19.542) | 0.649 | 1.664 (0.147-18.859) | 0.681 |
| Neonatal hypoglycemia | 1.075 (0.341-3.389) | 0.902 | 1.040 (0.328-3.298) | 0.947 |
| NICU Admission for > 24 hours | 2.130 (0.498-9.105) | 0.308 | 2.242 (0.521-9.639) | 0.278 |

*Adjusted for maternal BMI at booking visit


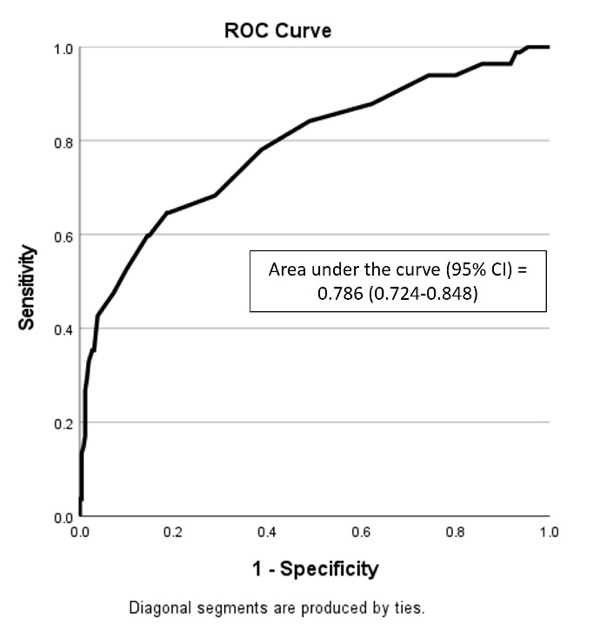

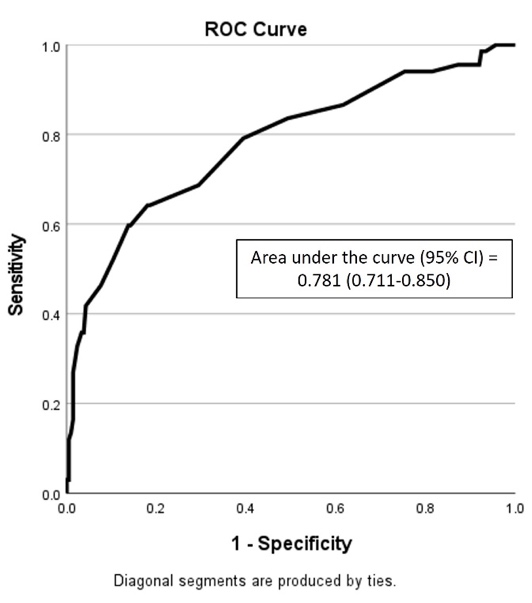


Supplementary Figure 1. a) ROC plot showing effectiveness of HbA1c in identifying diabetes in pregnancy as diagnosed by OGTT, b) ROC plot showing effectiveness of HbA1c in identifying diabetes in pregnancy as diagnosed by OGTT after exclusion of subjects with haemoglobinopathy, hemoglobin level < 11g/dL or MCV < 82 fL.
